# Graphene Quantum Dots as Flourishing Nanomaterials for Bio-Imaging, Therapy Development, and Micro-Supercapacitors

**DOI:** 10.3390/mi11090866

**Published:** 2020-09-18

**Authors:** Merve Kortel, Bhargav D. Mansuriya, Nicole Vargas Santana, Zeynep Altintas

**Affiliations:** Technical University of Berlin, Straße des 17. Juni 124, 10623 Berlin, Germany; mervekortel@gmail.com (M.K.); b.mansuriya@campus.tu-berlin.de (B.D.M.); n.vargassantana@campus.tu-berlin.de (N.V.S.)

**Keywords:** graphene quantum dots (GQDs), nanomaterials, bio-imaging, therapy development, micro-supercapacitors

## Abstract

Graphene quantum dots (GQDs) are considerably a new member of the carbon family and shine amongst other members, thanks to their superior electrochemical, optical, and structural properties as well as biocompatibility features that enable us to engage them in various bioengineering purposes. Especially, the quantum confinement and edge effects are giving GQDs their tremendous character, while their heteroatom doping attributes enable us to specifically and meritoriously tune their prospective characteristics for innumerable operations. Considering the substantial role offered by GQDs in the area of biomedicine and nanoscience, through this review paper, we primarily focus on their applications in bio-imaging, micro-supercapacitors, as well as in therapy development. The size-dependent aspects, functionalization, and particular utilization of the GQDs are discussed in detail with respect to their distinct nano-bio-technological applications.

## 1. Introduction

Graphene is an appealing substance with its sp^2^-hybridized extended structure, which was reportedly generated by mechanical exfoliation for the first time in 2004 and since then, it has been attracting considerable attention with its two-dimensional (2D) zero band gap and is well-known for its excellent electrical conductivity [1,2,3]. Graphene quantum dots (GQDs), being zero dimensional nanomaterials, are basically derived from the 2D graphene with their extremely small structure. While GQDs have extraordinary attributes coming from the quantum world, they also embrace carbon-derived characteristics like chemical and physical stability. This is particularly important since carbon is considered as the source of life from many aspects. The bio-applicability along with the properties inherited from the carbonic structure enables various opportunities for biological applications to these tiny, nanoscale particles [4,5,6,7]. GQDs are semiconductor nanomaterials that belong to the carbon quantum dots family [8], they display superior photo stability against blinking and photo bleaching [9], possess low toxicity, high biocompatibility [10], and good colloidal stability [11]. Owing to quantum confinement and edge effects, GQDs are holding extraordinary properties and attracting extensive attention [12].

GQDs exhibit much stronger photoluminescence (PL) than graphene sheets (GSs) [13]. In addition, their stability in terms of PL is preserved by resistance against photo bleaching and blinking attributes [14]. Although the PL mechanism of GQDs has not been fully explained, the assumptions about the PL source are strongly based on quantum confinement and edge effects [15,16].

GQDs exhibit unique fluorescence properties due to quantum confinement effect and possess a non-zero bandgap in the structure. The properties of GQDs can be tuned by modifying the size and shape by affecting the characteristics of their final structure [17,18,19,20]. Also, they possess peroxidase-like catalytic activity, which favors them in label-free biosensing [21]. GQDs have loads of negatively charged carboxyl groups and conjugated π–π bonds, which provide electrostatic properties for diverse applications [22], where the surface carboxyl groups bestow good water solubility to the GQDs [23]. The conjugation of other substances through the functional groups like carboxyl and hydroxyl groups found on the surface of GQDs can be facilitated [24]. The π–π stacking that possessed by the GQDs structure is also a good mediator for various conjugations of numerous substances [25,26,27].

The possibility of cellular internalization offered by GQDs is reportedly mediated by several different pathways including energy-dependent as well as clathrin- and caveolae-mediated endocytosis. Precisely, this internalization ability could endow the possibility for GQDs to target the cells and cell-organelles like endoplasmic reticulum, Golgi apparatus, and nucleus [28,29,30]. In addition, GQDs can attach the DNA via intercalation and electrostatic paths for the applications in bioengineering [31]. It was also shown that carboxylic groups found on the N-GQDs surface can bind single stranded DNA for the purpose of fluorescent biosensing [32].

GQDs are unique, valuable, and play a prime role in many fields like biosensing [33,34], drug delivery [35], solar cells [36,37,38], micro-supercapacitors [39,40,41], photodynamic therapy [42], and optoelectronics [43]. Although they play a pivotal and most important function in the development of biosensors, in this paper, our chief motive is to highlight their applications in the field of bio-imaging [44], micro-supercapacitors [45], and therapy development [46], since we have broadly covered their significance and detailed biosensing applications in our recently published articles [33,34].

### 1.1. Synthesis of GQDs

GQDs are tiny carbon-sourced materials and can be synthesized by bottom-up or/and top-down methods via diverse chemical synthetic approaches [47]. The top-down approach is mainly based on the physical cutting of GSs, where the bottom-up approach is based on the chemical synthesis of GQDs in pieces from small molecular fragments while enabling the management of their morphological properties [48,49,50]. Nanolithography, electrochemical scissoring of GSs, chemical exfoliation, and hydrothermal as well as solvothermal cutting of GSs are the methods for cutting approaches to obtain GQDs [16]. Improved Hummer’s method can be utilized extensively for the synthesis of GQDs, where the graphene oxide (GO) is the precursor of the quantum dot [51].

There are also some existing and well-established synthetic techniques offering advantages like smooth operation and low cost when synthesizing GQDs by direct carbonization of organic precursors such as amino acids, carbohydrates, ethylene diamine tetra-acetic acid (EDTA), di-thio-threitol (DTT), etc. While EDTA assisted N-GQDs, namely E-GQDs can be synthesized in a very short period of time, their characteristic analysis could reveal that these E-GQDs are the great candidates for opto-electronic and energy applications. In case of DTT yielded S-GQDs, 4-amino thio-phenol (ATP) could be a potent precursor for the nitrogen and phosphorous co-doped GQDs. It is worth noting that each precursor is treated at different temperatures in order to manufacture various heteroatom doped-GQDs [52].

Recently, Ji and coworkers developed a surface-assisted ‘bottom-up’ GQD synthesis method to successfully fabricate nanostructures smaller than 2 nm that enabled the quantum confinement studies [53]. A study showed that larger GQDs particles could be achieved by increasing the microwave heating time while utilizing the microwave-assisted hydrothermal method. The shorter lifetime was attributed to larger size of GQDs, resulted in lower quantum yield (QY) [54]. Likewise, the PL characteristics of GQDs was found to be pH-dependent [55]. Pan et al. developed a route of chemical reaction by proposing a simple hydrothermal approach to cut GSs into surface functionalized GQDs. Synthesized GQDs exhibited bright blue PL observed for the first time owing to the GSs and graphene nanoribbons (GNRs) because of their large lateral sizes [56].

Graphite is one of the most affordable and most readily attainable precursors for GQDs, and a study reported a facile method for synthesizing GQDs from graphite powders with a high yield and a chance of adjusting the emission of GQDs only by controlling system conditions during synthesis. Herein, the graphite was chemically oxidized at 40 °C to graphite oxide and the temperature of the system subsequently increased to 120 °C and left to reflux in order to cut the graphite oxide into small dots by an improved Hummer’s method that yielded crystalline GQDs in large scale [57]. Ye and coworkers achieved synthesis of GQDs from coal by proposing crystalline coal’s sp^2^ carbon structure as quantum dot source. Three different types of coal were used to derive GQDs; resultantly they have demonstrated different colors of fluorescence. Additionally, GQDs demonstrated solubility in water and held promising features for several bioengineering and electronics applications [55]. Another study involving the use of coal as the source of GQDs highlighted the antioxidant property of GQDs owing to their biocompatibility. Here, GQDs with oxidation sites were preferred for the antioxidant feature and these antioxidant GQDs were found to not possess fluorescence property, due to the modulation of reduction potential [58]. It is plausible to say that there are lots of GQD synthesizing methods which can be changed, modified, and improved with different approaches such as one-step solvothermal method [26], direct pyrolysis of citric acid [59], and hydrothermal fragmentation [60]. The important thing to pay attention is, each particular application demands a specific synthetic approach according to its necessity. Table 1 enlists various methods for the synthesis of GQDs using distinct sources with their reported sizes and corresponding QY.

### 1.2. Biofunctionalization/ Conjugation of GQDs

GQDs are being predominantly fashionable materials with their bio-applicable properties including eco-friendly nature [71]. They have been shown to be undeniably valuable throughout the research reported in recent decades. However, when functionalized in several ways, their properties and characteristics showed an improved trend line by encouraging fine-tuning and target-oriented applications.

The functionalization of GQDs prevents the aggregation caused by powerful π–π interactions among the GQDs [53] and cytotoxicity created by GQDs is confirmed to be dosage-dependent [72]. In a study based on the GQDs’ cytotoxicity, the mitigation of toxicity levels was developed on the hypothesis of whether the damage stemmed from the small sizes of GQDs or from their ability to create reactive oxygen species (ROS) intracellularly. Although the fewer amounts of GQDs do not necessarily create intracellular cytotoxicity, higher levels like more than 1 mg mL^−1^ result in cell deaths. Hence, the PEGylation of GQDs was employed to lessen the cytotoxicity that was thought to be a consequence of the decreased generation of ROS; thus the higher levels of GQDs utilization inside a cellular compartment was justified up to 8 mg mL^−1^ with 50% viability [73]. Another cytotoxicity study investigated the modifications of GQDs, and their contribution to the cell viability or lethality showed that even though GQDs hold varying groups like -NH_2_, -COOH, and -CO-N (CH_3_)_2_ on the structure, the low cytotoxicity phenomenon was preserved with no distinct changes proving the biocompatibility of the modified GQDs even at the concentration up to 200 μg mL^−1^. Results were assessed by the MTT assays performed on A_549_ lung carcinoma cells and C_6_ human neural glioma cells [74]. Another study investigated the contribution of size and concentration of the GQDs into cytotoxicity utilizing molecular dynamics simulations to reveal the structure and pristine GQDs’ translating mechanism at the molecular level, which showed that the smaller GQDs could permeate into the lipid bilayer, while bigger GQDs needed more time [75]. Moreover, it was also reported that the high concentrations of GQDs induced changes in the structural properties and diffusion characteristics of the lipid bilayer, and they may affect the cell signal transduction, revealing the fact that the cytotoxicity of GQDs with smaller size is low, which can be favorable for the bio-applications [75].

An early 2019 study, investigating the functionalization effects of GQDs on inducing cytotoxicity and autophagy of aminated (a-GQDs), carboxylated (c-GQDs), and hydroxylated GQDs (h-GQDs), made a consensus on the ability of autophagy and apoptosis. The h-GQDs showed distinctly more cytotoxicity comparing to the remaining two, whereas the c-GQDs was the most cyto-compatible among them, and was thus referred by the author as the “safest modification on GQDs” [76]. This is a good example that highlights the importance as well as the significance of synthesis and modifications of GQDs, which can be adjusted according to the aim of their respective application.

GQD-incorporated thin film composite membranes were composed to be utilized in forward osmosis (FO) desalination. A 2D sheet with 2.19 nm morphology of GQDs could make covalent bonding with polyethylenimine by forming stable GQD-incorporated membranes. In this scenario, GQDs could promote the water permeability and enhance the water-flux [77]. As far as the antibacterial properties of the “GQDs enhanced TiO_2_/Sb_2_S_3_ nanocomposite” against *Escherichia coli* (*E. coli*) and *Staphylococcus aureus* is concerned, the existence of GQDs in the nanocomposite could result in the generation of more electron and hole pairs, meaning more powerful antibacterial activity could be achieved with minimum inhibitory concentrations of 0.03 and 0.1, respectively [78].

As indicated previously, quantum dots (QDs) are small yet valuable formations. This smallness of the quantum dots gives them the unique optical properties like absorption and emission, thus resulting in better outshine than regular fluorescent compound and it is undeniable that GQDs become prominent due to their non-toxicity property, separating them from the other semiconductor QDs. Accordingly, this makes it easier and more reasonable to utilize GQDs in biological applications [79,80].

**Figure 1:**
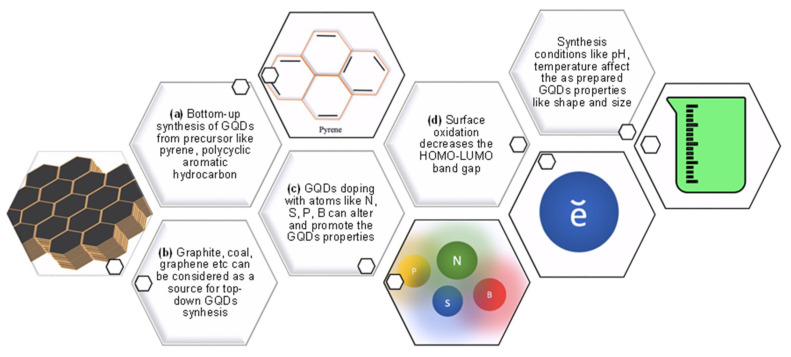
(**a**) Pyrene and polycyclic aromatic hydrocarbon along with other substances can be the precursor required for bottom-up GQDs synthesis [70,92]. (**b**) Several substances that can be the carbon source for top-down GQDs synthesis. (**c**) Doping graphene with heteroatoms, such as S, N, B, and P, can expertly manage a tunable gap in graphene’s energy spectrum and thus produce new phenomena, alter and improve properties of the dot [85,86,93,94]. (**d**) The size and the shape of the GQDs alter the energy levels of the highest occupied molecular orbitals (HOMO) and the lowest unoccupied molecular orbitals (LUMO) in correlation with the change of absorption spectra where the PL of GQDs are related to the passage from LUMO to HOMO levels. The band gap between HOMO-LUMO can also be decreased with the increase in surface oxidation of GQDs [95,96,97].

Since the first synthesis of chiral GQDs from Vázquez-Nakagawa’s group, the modification of GQDs has gained a new aspect. The synthesis of chiral GQDs was performed for the first time with the esterification with enantiomerically pure alcohol of (R) or (S)-2-phenyl-1-propanol where the chirality could be transferred to pyrene [81]. In addition, the utilization of GQDs on chiral separation was envisioned as a chromatographic separation agent by Wu et al. and further proved that the GQDs are appropriate agents for enantio-separation according to the comparison of the column efficiencies of β-cyclodextrin (β-CD) and cellulose silica composites both with and without GQDs. This study is also a good operation of molecular modeling in terms of revealing the dynamics between molecules and bringing clarification to why GQDs magnify the efficiency. According to the authors’ explanation, “GQDs provide extra interactions such as hydrophobic, hydrogen bond, and π–π interaction”, which makes this conjugation of GQDs plausible in the first place [82]. Moreover, the conjugation was also confirmed and employed by a sensor system that was designed to enantio-recognize of tryptophan isomers [83]. Additionally, it is possible to synthesize GQDs in combination with heteroatoms like nitrogen (N) [31,84,85], sulfur (S) [86,87,88], boron (B) [89], and oxygen (O) [90] which in turn, these dopings may enhance the capabilities of GQDs in varying manners. For instance, N-doped GQDs synthesized by a hydrothermal reaction could show high antioxidant property as compared to the conventional antioxidant agent utilized in food safety [31]. These N-GQDs hold an outstanding photocatalytic structural performance with considerably high QY [91]. A recently published study revealed loads of beneficial data about GQDs and their conjugation with hetero atoms like N and S. The N,S-GQDs, synthesized from citric acid and L-cysteine precursors can be conjugated with streptomycin antibiotic to develop drug conjugated QD for the improvement of drug efficiency, intracellular imaging and biocompatibility investigations. The presence of heteroatoms in GQD strengthens several prospects to promote the PL as well. The proposed N,S-GQD system was enriched by hydrophilic functional groups like -COOH, -OH, and -NH_2_ to provide solubility and drug conjugation [79]. In large scale productions of GQDs, their functionalization by sulfonation could provide re-dispersion in water and consistent fluorescence, and these sulfonated GQDs can be beneficial for intracellular Golgi imaging [28]. Figure 1 represents the summary of bio-conjugation of GQDs.

It could be suggested that GQDs can be a proper photodynamic therapy (PDT) agent due to their ability to create photosensitized oxygen. The study proposed by Jovanovic and the group revealed the vital effects of functionalization of GQDs when it comes to PDT applications. Introducing urea and thiourea, the N, S co-doped GQDs were synthesized as a better singlet oxygen generator compared to sole GQDs and accordingly, GQDs with urea were defined to be a good PDT agent. The modification with nitrogen could enhance the capability of GQDs by means of crystallinity and mediate more photo-excited singlet oxygen generation, resulting N, S- doped GQDs to be anti-oxidative [98]. 

### 1.3. Impact of Toxicity Potential and Biocompatibility of GQDs

The cyto-compatibility of GQDs results in tremendous biological applications and is already desirable for many cases. Yet, the probable cytotoxicity effects of GQDs on living cells should be considered concerning the biosafety [99]. Moreover, currently the researchers are investigating the best possible treatment and alternative approaches when it comes to human life; however, utilizing GQDs is a two-way road that should be evaluated carefully [100].

It has been well summarized by Zhang et al. that GQDs with small sizes and concentrations in the range of micrograms and milligrams were non-cytotoxic to mice and human cell lines. However, it should also be emphasized that several studies reported toxic effect of GQDs [101]. Wang et al. studied the cytotoxicity mechanism of N-GQDs and GO for the first time in 2014. Since the cytotoxicity was raised by the nonspecific adhesion of nano-sized particle to the cell membrane, the interaction between carbonic structures and red blood cells (RBCs) was evaluated. Accordingly, the N-GQDs were decided to be cytotoxic to the RBCs even they did not cause more serious effects than the GO [102]. Also, it was emphasized that the route of interaction between the living compartment and GQDs should be thoroughly investigated in order to obtain comprehensive information regarding the effect mechanism. On the other hand, PDT applications of GQDs are still pending for the detailed results regarding the cytotoxicity [103]. Another important aspect to toxicity of the GQDs is the excretion route of the GQDs after their application. As shown by Lee et al., GQDs were excreted from mice via renal pathway subsequent to their degradation by several enzymes like horseradish peroxidase (HRP) [104]. In addition, the degradation of GQDs by human myeloperoxidase and eosinophil peroxidase enzymes was also proven [105]. In order to decrease the toxicity caused by GQDs, PEGylation was suggested as a valid solution [73]. Another study conducted with the principle of PEGylation showed that the PEGylation may indeed lower the cytotoxicity since the GQDs themselves did not cause any remarkable cell death even at the concentrations of 160 µg mL^−1^ conducted in vitro with HeLa cells. In the same study, the comparison of GO-PEG and GQD-PEG was conducted in vivo which resulted in remarkable biocompatibility of GQD-PEG conjugation whereas three mice could not survive without any sign after the GO-PEG injection. The desirable biocompatibility was related with the small sizes and ease of clearance from their body and high oxygen content [106]. In a very recent study, it was demonstrated that GQDs can alter one gene’s expression on CD^34+^ hematopoietic stem cells after 36 hours of incubation, which is a negligible number within 20,800 genes [107].

**Figure 2:**
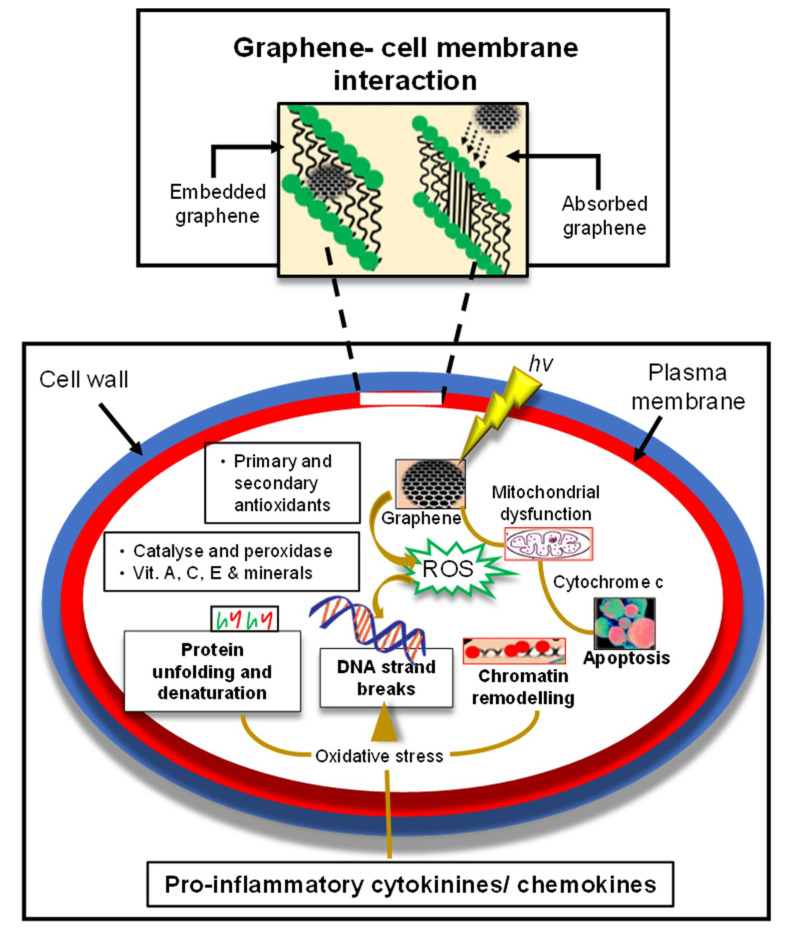
Schematic representation of the effective mechanisms by which ROS are linked with the toxicity of graphene at a cellular level. Adapted from [108].

As depicted in Figure 2, graphene also influences biologically at the cellular, subcellular, protein as well as genetic levels. The toxicity of graphene is based on its uptake in various particular organs as well as on its physical and chemical interference. Accumulation of graphene in such organs affects cellular performance. Their impeachment, dispensation, and excretion after entering in a cellular environment retrieve the information about their cyto-toxicity [108]. Mitochondria imaging has been achieved with several methods formerly, like making GQDs accumulate mitochondrial and lysosomal site by simple modification of aptamer AS1411 in order to label tumor cells. Yet the exact uptake mechanism of AS1411-GQDs remained unknown and was assumed to be similar as AS1411 [109]. Later on, when Fan et al. studied the GQDs-TPP (GQDs-phenyl bromide phosphine) as a targeted mitochondria imaging agent, they expected the lipophilic TPP to cumulate in the mitochondria for targeted imaging. Expectedly, nuclear and mitochondrial imaging was performed without any cytotoxic side effects [110].

It is an undeniable fact that the GQDs are being promising in numerous applications nowadays as aforementioned; however, it is an obligation to evaluate the adverse effects of GQDs in order to provide biosafety when translated into the clinical applications. Surface enhanced infrared absorption spectroscopy (SEIRA) has been very useful tool to assess the relationship between GQDs and their environment in the field of toxicology [102]. Table 2 outlines various forms of GQDs that are responsible for cytotoxicity.

## 2. Bio-Imaging Applications of GQDs

Bio-imaging is one of the most significant areas, where GQDs offer several benefits in an auspicious way [92,112]. In definition, bio-imaging is a way to see, observe, and detect the desired molecules or tissues in the body without performing any invasive application [113], which enables more comprehensive understanding of the biological pathways in the body [114]. When Wilhelm Roentgen could capture the first X-ray image in 1896 [115], since then, a new door has been opened for prospective bio-imaging applications to detect and monitor various diseases and symptoms like bone fractures [116], carcinoma [117], Parkinson’s disease [118], tumor imaging [119], etc.

GQDs are convenient and desirable agents in bio-imaging due to their admirable tunable PL characteristics, chemical inertness, high photo-stability, and excellent biocompatibility properties [64]. Moreover, GQDs hold superior properties compared to their counterparts of other carbon-derived materials [30] like noteworthy resistance to photo-bleaching that facilitates good bio-imaging purposes [110]. As a case in point, it was revealed that due to crystalline structure, GQDs are superior to carbon nanodots (CNDs) when irradiated with a Xenon lamp in terms of their bleaching activity and stability [97]. GQDs were proven to be stable particles during the imaging process without spoiling any results [28], and their fluorescence imaging with near-infrared (NIR) spectra is often encountered in the studies [66,120,121].

There are several other methods rather than solely utilizing GQDs in bio-imaging studies like organic dyes. However, the poor photostability, low QY, susceptibility to photobleaching, and unavailability for multicolor imaging characteristics are offered with these dyes [122]. On the side, QDs have been used for a while as imaging tools and are very well integrated into bio-imaging systems since they have overcome the challenges of conventional dying methods; however, later on, their toxicity became an issue for biological applications. In spite of toxicity caused by other semiconductor QDs, GQDs are justified for non- or minimum-toxicity features in biological tissues [74,75].

On the other hand, these GQDs could show elevated levels of illumination and photo-stability, along with their size-tunable properties [123,124]. Notably, the first demonstration of GQDs smaller than 10 nm in size could generate ‘bright-blue-PL’, different than GSs and graphene nanoribbons, started a new stage. This accelerated optoelectronic and labeling studies, as well as prospective GQD-utilized-bio-imaging applications. It is crucial to notice that strong luminescent emission of GQDs can be attributed to the dense, free zigzag edges found in the tiny structure stemming from <10 nm size. Also, it was shown that PL emission was highly dependent on the pH of medium; i.e., alkaline mediums could give the strong PL, whereas acidic media could not support it, suggesting that PL behavior can be reversed by altering the pH of the medium [56].

Plausibly, new methods that use GQDs for bio-imaging applications were quickly developed, owing to the desirable features of GQDs in terms of bio-applicability and non-toxicity compared to inorganic QDs [64,111,125]. As one of the first studies of GQDs for bio-imaging, Zhu et al. proposed a one-step solvothermal method for highly green-PL GQDs with an 11.4% QY, which emphasized that the GQDs can be employed as bio-labeling agents due to their bio-applicable characteristics [126]. With the time, the QY and conjugations of GQDs with other materials enabled better results in terms of their efficiency and utility. GQDs’ surface-modifications were shown to affect the PL [127], whereas photo-chemical reduction method was suggested for the increasing QY and elevated cell uptakes of GQDs [128]. GQDs’ surface-functionalization with small organic molecules proved to be an effective strategy for PL tunability through altering the band gaps, and decreasing the cellular toxicity [129]. Varied color emissions of GQDs were obtained with different synthetic approaches for bio-imaging aims. For instance, N-GQDs with blue luminescence were prepared by hydrothermal treatment of GO. Herein, 24.6% QY and biocompatibility of N-GQDs were high enough to illuminate HeLa cells, meanwhile having the blue-shift property with the increasing pH and temperature dependency influence the PL intensity [130]. Green luminescent GQDs can be synthesized by a facile synthesis method in large-scale from graphite powder for imaging human hepatic cancer cells [131]. Later on, water-soluble, uniform-sized GQDs with red fluorescence (RF-GQDs) demonstrated a high bio-imaging applicability, through their dominant red color as a robust biological marker for stem cells [63].

It was shown that the green synthesis of GQDs could overcome the cytotoxicity due to their biocompatibility as well as their excellent sized-tuned emission features [125]. A study that employed mango leaves as the source for GQDs showed total cellular uptake and cell viability, even at high concentrations, for 24 h of GQD treatment while showing NIR emissions between 650–750 nm. This NIR excitation-independent fluorescence emission could satisfy the need for far penetration into tissues required for in vivo administrations [66]. In the course of cytotoxicity, recently, Yan and coworkers proposed a highly biocompatible GQDs (HGQDs) synthesis method by only employing glucose in the absence of any acids or oxidizers. HGQDs were synthesized via hydrothermal one-pot method by autoclaving the glucose solution at 200 °C for 10 h. In contrast to conventionally synthesized GQDs (CGQDs), HGQDs possessed diminished levels of apoptosis with almost 60% reduced cytotoxicity and 2.24% QY, exhibiting superior cyto-compatibility compared to the conventionally synthesized GQDs owing to glucose precursors. The as-prepared HGQDs were shown to have a similar structure to glucose, which in turn could enhance the cellular uptake of these small dots due to such similarity. HGQDs were accumulated in the liver, kidney, and brain of mice, revealed by ex vivo tests. Moreover, it was examined that cancerous cell viability was increased by sugar based HGQDs. Cancer cell imaging showed that CGQDs could damage the cancerous cells when interacted, whereas the HGQDs could bestow better cytocompatibility for bio-imaging. Likewise, bacterial imaging studies supported the earlier results of CGQDs’ shape-destructive characteristics compared to HGQDs. When ex vivo imaging was performed with the isolated organs of mice, which were already treated with HGQDs for 20 days, the liver, kidney, and brain confirmed to possess HGQDs (Figure 2). Their accumulation particularly in the brain indicated that the as-prepared GQDs can pass through the blood–brain barrier. This approach may facilitate the prospective brain-related studies for GQDs-imaging [10].

In a 2017 study, as a novel application, Chen and coworkers could successfully functionalize GQDs with sugar moieties, referred to “sweet GQDs” that were used for real-time monitoring of specifically labeled carbohydrate receptors to pursue their real-time dynamics. The synthesized GQDs had almost QY of 31% and small sizes of 5.4 nm. Consequently, since the investigation of some overexpressed receptors in tumors could help the identification of certain cancerous cells, the galactose tagged GQDs (Gal-GQDs) could specifically interact with overexpressed galactose receptors in the liver and fluorescently lighten them up. Likewise, mannose receptors were overexpressed in human breast cancer cells, and Man-GQDs could selectively identify mannose receptors found in the several body parts [92].

Magnetic resonance imaging (MRI) is one of the numerous imaging tools that exist to allow the clinical diagnosis along with other techniques like positron-emission tomography (PET), computed tomography (CT), fluorescent and photoacoustic imaging, as well as medical optical imaging [132,133]. As one of the most advantageous imaging methods, with its gadolinium oxide (Gd_2_O_3_) T1 (positive) contrast agents, MRI technique can be coupled with the two-photon fluorescence imaging (TPFI) GQDs. Wang and coworkers offered an excellent and MR suitable contrast agent, i.e., GQD-conjugated Gd_2_O_3_. Owing to the abundant edge functional groups on GQD layers, colloidal stability and biocompatibility of nanocomposites in water, it was reported that Gd_2_O_3_/GQD could exhibit tremendous optical properties, where the MR relaxivity (15.995 mM^−1^ s^−1^) proved that Gd_2_O_3_/GQD could serve as an MR T1 contrast agent. Moreover, both one-photon and two-photon diagnostics were possible with the proposed agent [134].

The importance of the TPFI is stemmed from the fact that it can perform non-invasive tissue penetration and can minimize the cytotoxicity when interacted with tissues [135,136,137], which make them advantageous for bio-imaging. The TPFI enables more extended detection time and reduced photo-damage for the tissue [138]. The metal-free boron-doped magnetic GQDs (B-GODs) were shown to display sustainable behaviors convenient for TPFI and near infrared (NIR) imaging contrast agent. Wang and coworkers proposed B-GQDs as a safe T1 contrast agent for MRI, due to the ferromagnetic property of metal-free (MF) graphene since Gd-based contrast agents could model some safety concerns. Henceforth, the suggested 5 nm sized B-GQDs demonstrated a paramagnetic property without any metal employment, enabling the potential service as a contrast agent for T1 MRI [23].

**Figure 3:**
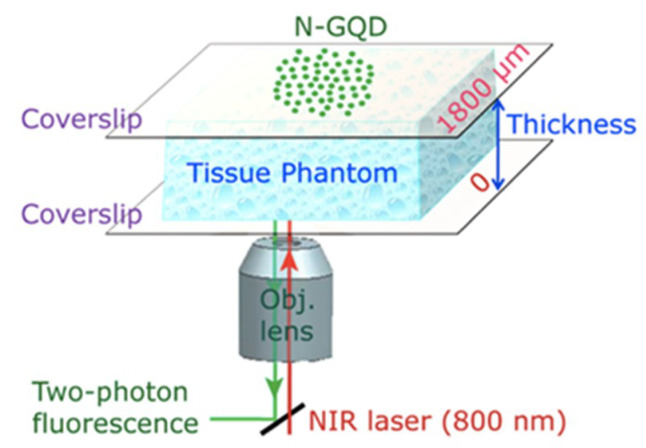
Schematic representation of the set-up used for TPFI of N-GQDs in tissue phantom with different thickness. TPFI is known for deep penetration into tissue with mitigated photo-damage during application. TPFI of N-GQD was analyzed using a NIR femtosecond laser as excitation and implemented for tissue imaging up to the penetration depth of 1800 μm. With negligible photo-thermal effects of N-GQDs, it was possible to use N-GQDs in TPFI on living cells with strong fluorescence [139].

**Figure 4:**
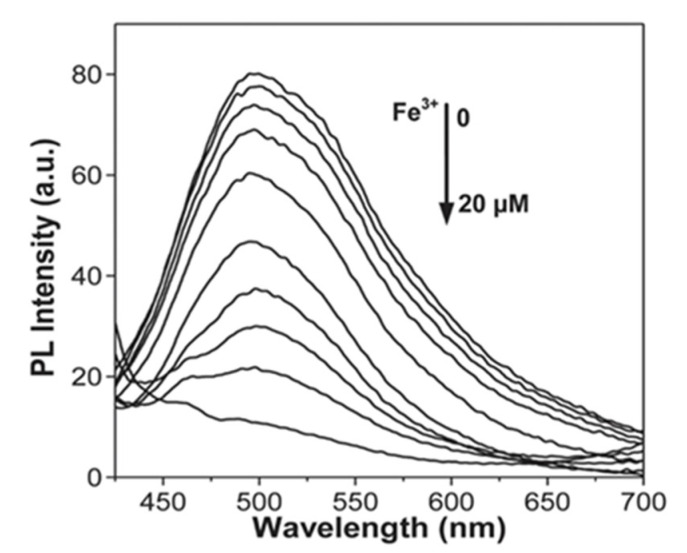
PL intensity changes due to the Fe^3+^ application on the quenching mechanism (λ_ex_ = 400 nm). Fe^3+^ ions are indispensable, and it was hypothesized that GQDs can be a detector of Fe^3+^ since they have phenolic hydroxyl groups. These results indicate that luminescent GQDs can also be used to detect Fe^3+^ [70].

N-GQDs were demonstrated as a TPFI probe on the intra-lipid mock tissue and resulted a good bio-imaging quality. Additional to the aforementioned properties of GQDs, a lack of thermal damage caused by the heat of irradiation characteristics of the proposed GQDs showed deep tissue penetration up to 1800 μm (Figure 3) [139]. Moreover, in a study that employed both the bio-imaging and biosensing experiments executed with the large-scale-synthesized GQDs were efficiently internalized by the MCF-7 cells and generated a strong bio-imaging capability. As the sensing unit, the existence of Fe^3+^ ions in the medium quenched the fluorescence (Figure 4). These polycyclic aromatic hydrocarbon derived GQDs measuring 5–10 nm were shown to be an excellent bio-imaging probes with features such as solubility, PL, low toxicity, and stability [70]. Another study related to Fe^3+^ sensing with GQDs inferred that rhodamine B functionalized GQDs (RBD-GQDs) pair can indeed sensitively detect Fe^3+^. Here, Guo et al. showed that Fe^3+^ detection resulted with a peak at 559 nm. The authors claimed that a ring opening phenomenon was occurred via Fe^3+^ in the RDG structure. However, in contrast to the previous results where the Fe^3+^ detection led to the decreased PL, the results of this study showed that as the Fe^3+^ increases, the fluorescence intensity increases where the saturation point is 22.5 μM of Fe^3+^ (pH 7.0) [140]. Another study related to this specific Fe^3+^ detection in an acidic medium demonstrated that at pH 3.5 without any special treatment, GQDs can specifically detect Fe^3+^, even in the co-existence of other ions. However, the increasing pH value showed the interference from other ions and selectivity for Fe^3+^, which was quite low at pH 1.4. Moreover, this study showed similar results in terms of quenching performed by Fe^3+^ [141].

**Figure 5:**
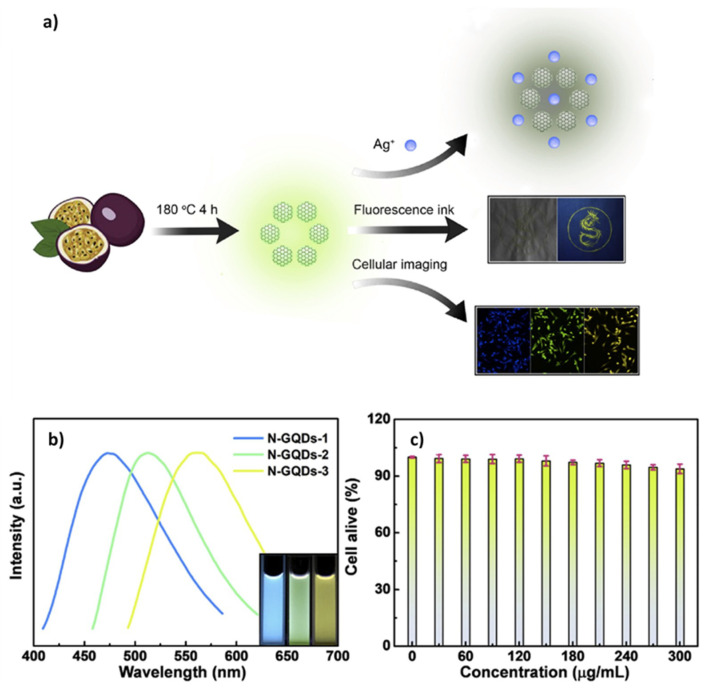
(**a**) Representation of the synthesis of N-GQDs from *Passiflora edulia* Sims, followed by the biosensing, ink, and bio-imaging applications. (**b**) The mechanism for the multicolor emissions of the N-GQDs was performed under different wavelengths. Blue, green, and yellow emissions were obtained under 472, 509, and 565 nm, respectively. (**c**) The correlation between the increasing amounts of N-GQDs and fibroblast cell viability [143].

A Cr(VI) biosensing and bio-imaging system were submitted on the MCF-7 cells as well. The biosensor system was adjusted for the detection of Cr(VI) even under running water, with a limit of detection (LOD) of 91 nM. The N-GQDs were synthesized by a one-step hydrothermal method with a 64.2% QY. N-GQDs were proven to be an exceptional in terms of their stability. As a fluorescent probe, the quenched N-GQDs were recovered by adding Na_2_S_2_O_5_ and FeSO_4_. The imaging studies performed on MCF-7 cells showed that N-GQDs can be utilized for further bio-imaging activities since these particles were internalized via endocytosis and could produce a blue-PL under 405 nm excitation light [142]. Likewise, in a recent study, a biomass-derived N-GQDs were hydrothermally synthesized and employed as fluorescent inks. Herein, the final structure of N-GQDs could exhibit the blue, green, and yellow emission under 472, 509, and 565 nm, respectively. The bio-imaging part was demonstrated as a multicolor PL system, where it had a 29% of QY that resulted due to their desirable bio-applicability properties. Having exhibited higher stability under irradiation, the resistance against photobleaching of N-GQDs was proved and shown to be non-toxic for the fibroblast cells (Figure 5). On the other hand, the prepared N-GQDs were illustrated to be greatly quenched by Ag^+^ ions, indicating the biosensing activity against this particular ion. In this case, the Ag^+^ ion detection was enabled via N-GQDs with an LOD of 1.2 nM via ‘turn-off’ principle. The quantification of Ag^+^ ions in water samples is significant for environmental analysis, since excess Ag^+^ ions may cause toxic effect in skeletal system and liver as indicated by the World Health Organization (WHO) [143]. Due to this reason, it was shown in sensing experiments that N-GQDs were extremely sensitive for the Ag^+^ ions compared to the other metal ions. In this study, the PL intensity was in correlation with the pH levels of the medium where the optimum quenching value was obtained at pH 7.0 while the different wavelengths generated multicolor emissions to enable cell imaging. Moreover, when N-GQDs were incubated with fibroblast cells, it was observed that there was almost no toxicity in the system [143].

**Figure 6:**
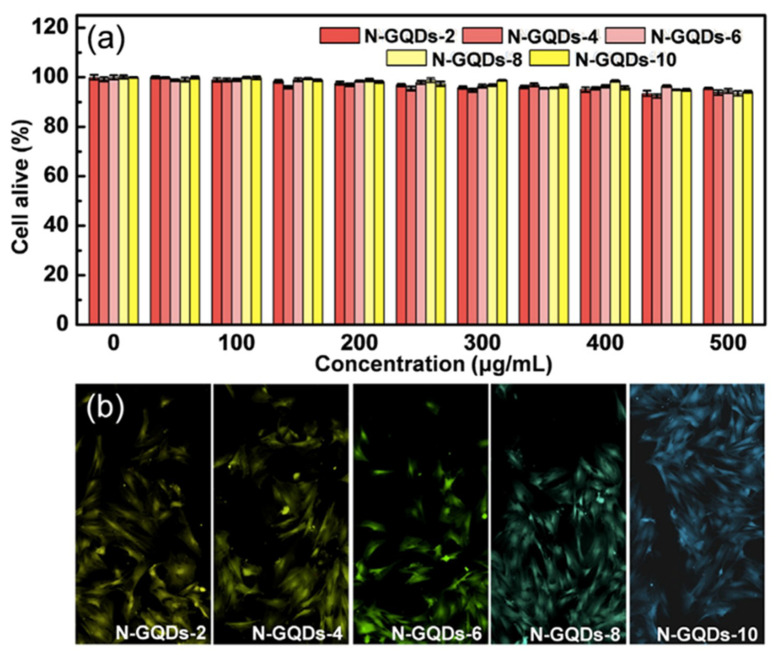
(**a**) Representation of concentration versus cell viability relation. Different reaction times (from 2 h to 10 h indicated as N-GQDs-2 to N-GQDs-10, respectively). (**b**) Confocal images of N-GQDs (100 µg/mL) incubated with fibroblast cells, indicating the internalization with different colors of emission [93].

Another N-doping study conducted with GQDs was recently published on the subject of controllable doping of GQDs [82]. The doping was performed with dimethylformamide via UV radiation at room temperature, where the doping concentration was changed upon the reaction time. Hence, as a result, GQDs were confirmed to have different QY based on their varying reaction times and doping concentration. The increasing doping of GQDs elevated the QY and provided a blue shift. The emission wavelength was shown to be tunable from 566 to 463 nm with the increase in reaction time. These N-GQDs were also nontoxic in vitro and produced great PL inside the fibroblast cells. Like the former studies with no obvious and significant cell death, the biocompatibility of GQDs was re-validated (Figure 6a). In conclusion, the N-GQDs were internalized by the cells as shown in Figure 6b, where the controllable doping of GQDs was demonstrated [93].

The pentaethylenehexamine (PEHA) and penicillamine (DPA) co-functionalized GQDs, namely PEHA-GQD-DPA, as a fluorescence probe with 90.91% QY have been successfully applied in the bio-imaging of Hg^2+^, which is one of the most prevalent heavy-metals that threatens human well-being. PEHA-GQD-DPA probe was shown to have an augmented fluorescence emission owing to their co-functionalization with PEHA and DPA and stated substantial photostability. Moreover, the existence of Hg^2+^ with PEHA-GQD-DPA, resulted in fluorescence quenching. This approach could qualify the optical detection of Hg^2+^ in natural water. The fluorescence response of Hg-PEHA-GQD-DPA complex towards various molecules, including glutathione (GSH), was studied and determined that GSH could generate an increased fluorescence intensity of Hg-PEHA-GQD-DPA complex. Consequently, the bio-imaging experiments conducted on SW_480_ cells showed that PEHA-GQD-DPA could image intracellular Hg^2+^ and GSH ultimately, where it was shown that Hg^2+^ could quench the fluorescence of the complex while GSH could well recover it. In this manner, the one by treatment of these substances with PEHA-GQD-DPA stained cell could enable the sequential detection of Hg^2+^ and GSH [62].

**Figure 7:**
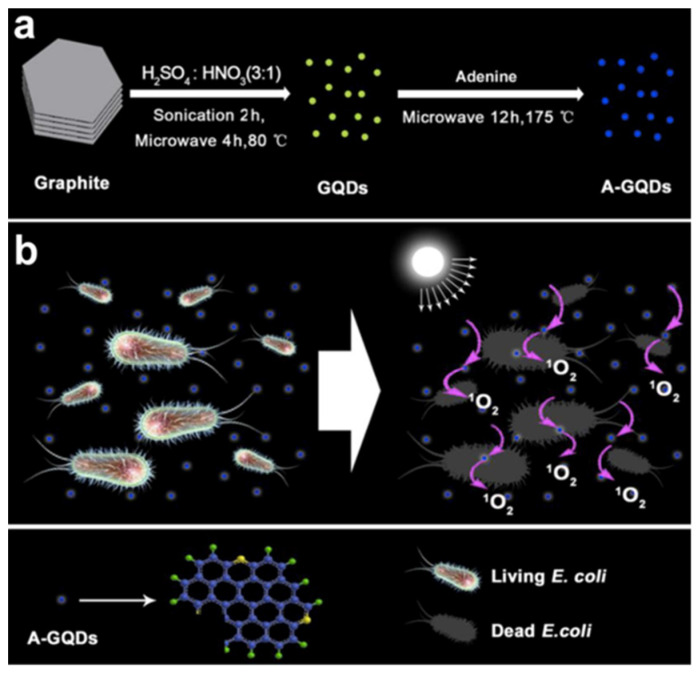
(**a**) Schematic illustration depicting the microwave-assisted preparation of A-GQDs. (**b**) White light-activated antibacterial property of A-GQDs towards *E. coli.* GQDs aqueous solution and adenine were reacted in a quartz tube for 12 h in the microwave reactor at 175 °C and left for 12 h at 4 °C, subsequently filtered by a microporous membrane to remove the residual adenine. Two-day dialysis was performed to obtain a pure colloidal solution. After evaporation, A-GQDs were obtained for the further use. For the first time, it was shown that the adenine modified GQDs also own white light-activated antibacterial features along with their high fluorescence ability with a QY of 21.63% at 350 nm [61].

The bio-imaging activity of GQDs can also be considered along with their antibacterial property. In 2017, a study on adenine modified GQDs (A-GQDs) was reported, claiming for the first time that the synthesized QDs can be employed for the antibacterial applications under white light. Herein, A-GQDs were prepared by blending the bulk graphite and adenine (Figure 7). The synthesized A-GQDs were reported to be an exceptional in cell imaging due to their green TPFI property at an excitation wavelength of 750 nm. In this study, the human lung carcinoma A_549_ cells were selected as a test model, where the A-GQDs were shown to have high-grade biocompatibility and resulted into the cell viability at 94% after 24 h of incubation, which proved that A-GQDs could be the appealing candidates for further bio-imaging applications. The white light-activated antibacterial activity of A-GQDs against gram-positive *S. aureus* and gram-negative *E. coli* showed that there was nearly no significant proliferation and vital activity of *E. coli*, however *S. aureus* could stay alive without being affected by A-GQDs [61].

**Figure 8:**
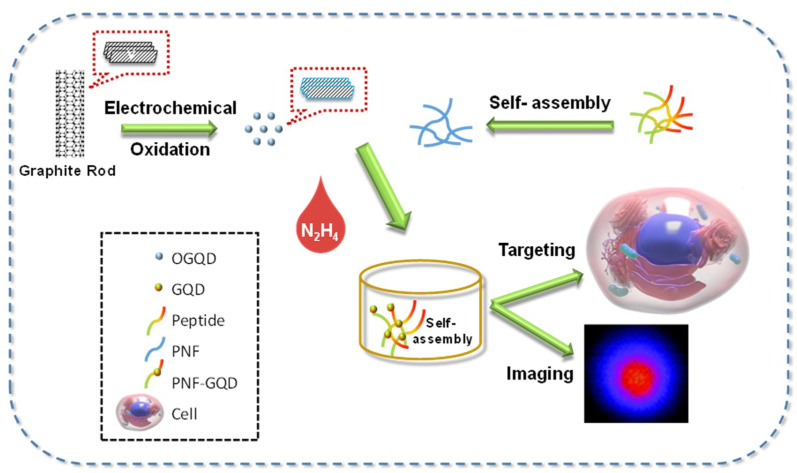
Schematic representation of the study in brief. The graphite rods were electrolyzed to the oxidized GQDs (OGQDs) and GQDs were synthesized by hydrazine hydrate reduction from OGQDs. Highly crystalline GQDs had 2–3 graphene layer with 0.25 nm lattice spacing. The peptide was designated to have AEAK sequence (for the self-assembly), RGD sequence (bind to integrins of the tumor), and YWYAF sequence (can bind to graphene). Subsequent to self-assembly, the PNFs were obtained with the height of 0.2–0.5 nm. Afterward, PNF–GQD nanohybrids were formed and were ready for the further experiments in targeting and bio-imaging.

It was presented that various strategies could be developed for specifically targeting cells, or tumors in imaging applications [144,145,146]. Employing GQDs as imaging probes conjugated with targeting sites could be a good strategy for the cell-targeted bio-imaging applications. For instance, Su and coworkers designed a novel nanohybrid structure for concurrently visualizing and targeting cancer cells by taking full advantage of the optical properties of GQDs and the specific recognition capability of arginine–glycine–aspartate (RGD)-containing peptide nanofibers (PNFs). Since the RGD sequence could accurately recognize the integrin-rich solid tumor type, the labeling of tumor cells was successful. The biocompatibility test of the GQDs and RGD-GQDs compared to the control group generated a very promising result with substantial cell viability even after 72 h. Moreover, it was hypothesized that the PNFs can be advantageous for cell reproduction, since the cellular viability was increased when incubated with PNF-GQDs (Figure 8) [11].

Progressively, another study published in 2018 also employed the RGD conjugated GQDs through π–π stacking (RGD-GQDs) for the loading of doxorubicin (DOX), an anticancer drug, besides aiming for the cancerous cell imaging along with the monitoring of drug activity in the body. As aforementioned, the specific recognition ability of RGD along with excessive optical properties of GQDs make their conjugates the desirable candidates in targeted imaging studies. Moreover, by loading the anticancer drug DOX into this existing combination, a cancer-cell-destructive targeted cell delivery complex was constructed. In this case, it was advantageous that cancerous cells have lower pH values than the normal healthy cells, since the DOX released from the DOX-GQD-RGD complex could show a powerful trend in an acidic media. At the end of 72 h the most DOX release was significantly higher in the acidic media. The cytotoxicity results performed with U^251^ human glioma cells showed that GQD does not have a significant lethal effect, whereas both the DOX-GQDs-RGD and GQD-DOX lethal activity were superior to plain DOX treatment in glioma cells. This study also emphasized the potential of GQDs to be the pH-responsive drug carrier vehicles [65].

That GQDs can get along with biological cells is a very important feature even though every study is not directly built for the diagnosis or in vivo imaging. This good relationship has facilitated the primary bio-imaging applications and is still being challenged by the novel methods and studies. It is critical that the internalized substance for the imaging should be ideally non-toxic for the cell, otherwise the cytocompatibility would hamper the bio-imaging applications. Thus, a combination of GQDs with different materials employed for various applications must be biocompatible. In this case, polyethyleneimine (PEI) conjugated GQDs can be a good example. The embedding or coating of GQDs with PEI provided separate colors to these particles stemmed from the structural difference in a non-toxic way for the human embryonic kidney and human primary glioblastoma cell lines. PEI attached GQDs formed the PEI_1800_ and embedding GQDs into PEI formed PEI_600_ materials that produced blue and red emissions, respectively. Uncoated GQDs emitted yellow light and could finely disperse in water along with the other GQDs. The PL-tunable properties with red and blue shifts were obtained by the interaction of PEI with GQDs [147]. However, this PEI and GQDs application does not exhibit any limitation, instead this can be adjusted for other imaging applications.

**Figure 9:**
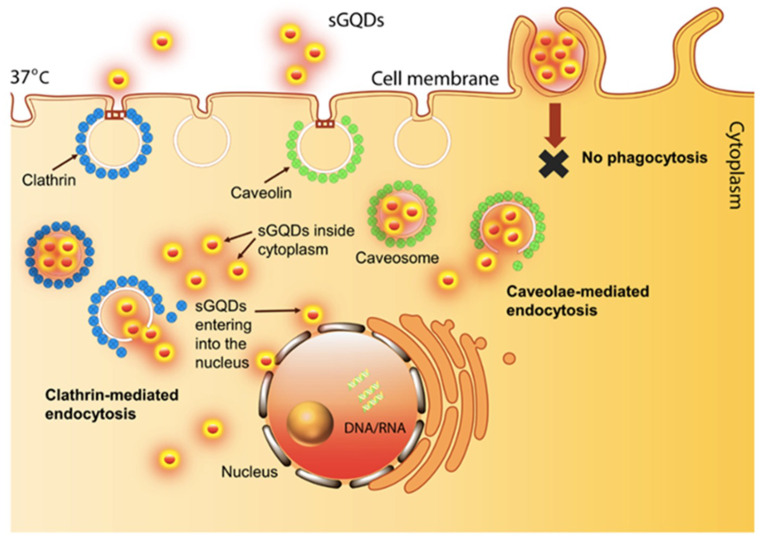
Nucleus labeling was performed when sGQDs were directed towards the cell nucleus by themselves. The figure represents the cellular and nuclear uptake of the sGQDs by endocytosis mediated by clathrin and caveolin endocytosis inside the cell. The uptake mechanism shows the localization of sGQDs inside the nucleus. Even though the sGQDs could not bear any nuclear localizing agent, according to the previous studies, their small size, surface charge, histone-chaperones, and nuclear-localization signals execute localizing inside the nucleus. Thus, the nucleus imaging and potential drug delivery applications with sGQDs are more likely to happen with their eco-friendly features. In addition, with no ROS generation, this nucleus staining method is confirmed to be biocompatible and non-cytotoxic [30].

In 2019, a different GQD-PEI study was conducted along with the phenyl bromide phosphine conjugation with GQDs (GQD-TPP). This study aimed to monitor targeted parts of the cell, where the GQD-PEI was expected to image the nucleus. Superior properties of GQDs facilitated the bio-imaging activity since GQD-PEI could perfectly target and label the cell nucleus. GQD-TPP was arranged for mitochondria imaging and similarly applied to mitochondria. The GQD-TPP viability was shown to be higher than GQD-PEI counterparts though both subjects were confirmed to be cyto-compatible. Thus, the low toxicity, low-cost properties when combined with photostable GQDs, this substance was recognized as a great candidate for prospective cell targeting and monitoring agents [110]. Another strategy to construct a bio-imaging platform for cell nuclei was proposed by Kumawat and coworkers. Herein, self-assembled GQDs (sGQDs) were bio-synthesized from grape seed extract (GSE) with a QY of 31.79%. In the proposed method, sGQDs were prepared by dissolving grape extract in absolute ethanol and dried subsequent for the filtration. The self-assembly was formed by electrostatic interactions that occur between functional groups of nanomaterial and solvent, which was critical since these sGQDs could enter the cell nucleus due to their small sizes. Expectedly, sGQDs and GSE were biocompatible and non-toxic for the L_929_ cells as there was no ROS generation. In vitro scratch assays showed that sGQDs indeed have proliferative cellular activity. Moreover, sGQDs were managed to enter fibrosarcoma, pancreatic cancer, ovarian cancer, and osteosarcoma cell lines, and they self-localized themselves inside the nucleus of these cells (Figure 9). As a result, this phenomenon demonstrated that, sGQDs are advantageous in terms of the superiority they show over other nucleus dyes because these dyes are not highly photo-sensitive, besides they are difficult to be handled as well as expensive. However, these green-synthesized sGQDs can uniquely image nuclei while being highly effective with a small surface to volume ratio and self-localization characteristics inside a nucleus [30].

Lately, GQDs mediated bio-imaging applications were being elevated due to their significant bio-applicable properties. Instead of only offering in vitro observation of the luminescence they exhibit, the experiments conducted in vivo are being executed and are the elaborative and promising site of the imaging applications. Moreover, these in vivo studies can be aimed for the imaging of targeted parts of the body. Liu et al. prepared the europium–GQDs complex with dibenzoylmethane (DBM) and 1,10-phenanthroline (Phen) that demonstrated red fluorescence with high water solubility, 15.5% QY, and high color purity. Due to the enhanced permeability and retention (EPR) effect, the as-prepared complex was expected to stain tumor site with its bio-imaging capability. The in vivo and ex vivo experiments showed very good PL in the tumor site when interacted with the europium–GQDs complex. However, it should be also noted that, rather than only tumorous site, other organs were also weakly luminescent. These results are promising in terms of diagnosis and rapid intervention to the tumor site in the body. Nevertheless, such studies are speculated to be developed in the near future for particular and precise applications [64].

Due to EPR effect, the accumulation of nanosized particles into the tumor site is expected. The endothelial layer is weakened around the tumor tissue and vascularization occurs densely while retaining the macromolecular structures [148]. It is plausible to take advantage of EPR while designing anticancer systems. On the other hand, actively targeting the tumor folate receptors with folic acid (FA) is an alternative approach to tumor targeting for therapeutic purposes which is now becoming more and more prevalent. It was shown by various studies that FA can selectively target folate receptors found on tumor cells [149,150,151]. Hence, the conjugation of FA and PEG with GQDs along with ^131^I justified the tumor cell targeting and single-photon emission computerized tomography (SPECT) imaging by Wang and coworkers in 2019 [152]. According to their findings, PEG prompted the biocompatibility of the GQDs, and FA could target the folate receptors. The complex of ^131^I-GQDs–PEG–FA was safe for experimental conditions. Moreover, this complex can be metabolized in two days and can be excreted by urination. This accounted for an efficacious radioactive imaging probe for the tumor cells.

So far, the lab-scale production of the GQDs for the target aimed applications was the focus of interest. However, it is also possible to design large-scale-fit GQDs for industrial purposes. In 2013, a study suggested that large scale GQDs can be managed to be synthesized by an improved Hummer’s method. Even though the as-prepared GQDs showed low QY like 1%, the good biocompatibility on the A_549_ cells along with hydrothermal therapy for the QY was promising for further studies [57]. Later on, other methods revealed the large-scale synthesis of GQDs for various purposes [153,154,155]. However, in 2016, Wang et al. introduced the large-scale production of GQDs based on the carbon amounts of rice husk biomass. Just like the most of the GQDs, these as-prepared particles also demonstrated great biocompatibility when tested with HeLa cells along with blue PL [156]. In the same year, high defect- (HD-GQDs) and low defect-GQDs (LD-GQDs) were synthesized by exfoliation in order to be used in bio-imaging as fluorescence probes. Cell imaging investigated with the fibroblast L_929_ cells showed that the GQDs were blue luminescent when exposed to 365 nm irradiation and proved to be potential visualization tools with substantial biocompatibility [67]. In 2017, Wang et al. produced high quality sulfonated-GQDs at an industrial-scale for bio-imaging the Golgi apparatus. The significance was due to both high-quality and particular Golgi imaging rather than whole cell imaging [28]. A study that employed both GQDs and CNDs showed the different PL properties of these dots. GQDs did show two characteristic absorption peaks and CNDs had only one; where the GQDs had higher stability and low photobleaching property. The industrial scale mass production was supported by the convenient room temperature synthesizable characteristics of these carbon derived dots with toxic-free nature and high QY of 17.5% for GQDs and 35.3% for CNDs. Since the HEp-2 cells showed staining property when interacting with these carbon dots, the bio-imaging applications were also revealed to be possible by this study [97].

## 3. Role of GQDs in Therapy Development

Amidst some graphene-based nanomaterials such as GO, fullerenes, carbon nanotubes (CNTs), GQDs show a significant potential for therapy development in nanomedicine and biotechnology [157,158]. Their exceptional properties discussed in the earlier section render them the new promising tool for biosensing [89], biomedical imaging [63], drug delivery [46], and photo-thermal and photodynamic therapy [42,98].

**Figure 10:**
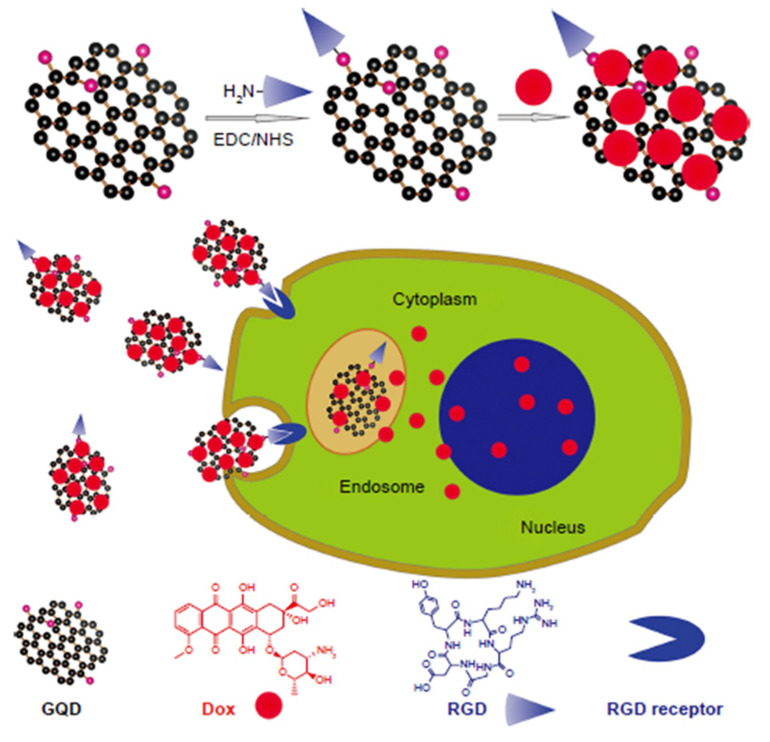
Schematic representation of multifunctional GQD-based DDS for the targeted delivery of DOX and their interactions with prostate cancer cells [166]. DOX, doxorubicin; RGD, arginine-glycine-aspartic acid; EDC/NHS, 1-(3-(dimethylamino)propyl)-3-ethylcarbodiimide and N-hydroxysuccinimide.

As previously mentioned, GQDs are arousing a great interest in the application of drug delivery, specifically for anticancer therapy and Alzheimer’s treatment. Drug delivery systems (DDS) transport the pharmaceutical compound into the human body in order to fully achieve their medicinal effect [113,159,160,161]. GQDs have been used as drug carriers by taking advantage of their π–π interactions, their small size and their ability to conjugate their surface with targeting ligands [162]. Moreover, functionalized GQDs have shown substantial efficiency on recognizing cancer receptors, selectively delivering chemotherapeutic agents, such as doxorubicin (DOX) or cisplatin, to the cell nucleus while enhancing their cytotoxicity, hindering their incorrect distribution into normal cell tissues and preventing drug resistance [163,164]. Liu et al. proved the viability of producing a multifunctional DDS (Figure 10) using fluorescent GQDs covalently linked with targeting arginine-glycine-aspartic acid (RGD) peptides for the enhanced delivery of DOX into prostate cancer cells [165]. Furthermore, many studies have confirmed the improved bioavailability of GQDs and the increased nuclear accumulation as well as DNA fragmentation activity of the therapeutic drugs [166]. Zhou et al. reported the good embedding ability of small-sized GQDs toward DNA cleavage [167]. These GQDs were synthesized from GO using a Fento reagent (Fe^2+^/Fe^3+^/H_2_O_2_) under UV irradiation and their wide applications were further investigated using copper ions (Cu^2+^) in a DNA cleavage system. This research group proved that by using GQDs and Cu^2+^ a significantly higher amount of DNA could be modified into non-continuous DNA than by employing GO and Cu^2+^. Presumably, the small lateral size of GQDs allowed them to easily insert themselves between the DNA molecules, causing the aforementioned cleavage.

Amyloidosis is a well-known aggregation and deposition of amyloid proteins as cross-beta sheets or fibrils in plaques around cells leading to organ and tissue failures. This phenomenon is responsible for a great number of degenerative disorders such as Alzheimer’s disease, Parkinson´s disease, type 2 diabetes, as well as for providing stability to bacterial biofilms [168,169,170,171,172,173]. Therefore, various candidates for the inhibition of amyloid aggregation, such as small molecules and peptides, have been studied over the years [174,175]. Protein accumulation inhibitors generally try to substitute the hydrogen bonding, hydrophobic interaction, and π–π -stacking of the amyloid protein to disassemble their aggregation [173]. GQDs have been reported as outstanding inhibitors of aggregation, toxicity of pathogenic and functional amyloid species due to their functionalized surface, which improves their antiamyloid targeting and delivery [175].

Alzheimer’s (AD) and Parkinson’s diseases (PD) are two of the most common chronic neurodegenerative disorders, which are gradually responsible for the deterioration of both the mental as well as physical activities of a subject, respectively. These disorders are characterized by the aggregation of ß-amyloid (Aß) and a-synuclein (a-syn) peptides respectively, causing extracellular amyloid plaques in the brain [169,176]. Due to some therapeutic deficiencies—such as low in vivo stability and efficacy, negligible permeability in the blood–brain barrier, and complex fabrication—many aggregation inhibitors for Aß and a-syn peptides have been ruled out as non-beneficial biomedical agents for either AD nor PD treatment [177,178,179]. Liu et al. presented the efficiency of using GQDs as a low-cytotoxicity inhibitor for the agglomeration of Aß peptides as they attach themselves to the hydrophobic center of the peptides [180]. This work demonstrated the possible biomedical application of GQDs as an anti-AD treatment due to their quantum confinement effect, edge effect, and remarkable biocompatibility. In addition, Kim et al. reported the advantages of GQDs as an anti-aggregation agent for PD by directly interacting with mature a-syn fibrils leading to their fragmentation [179]. Human islet amyloid polypeptide (IAPP) is another acclaimed amyloid protein that can be triggered by many biological, environmental, and chemical factors into ß-sheets structured agglomerations and is also a distinctive mark of type 2 diabetes [181]. Wang et al. exhibited GQDs as an efficient candidate for the prevention of IAPP accumulation and toxicity in an embryonic zebrafish due to their electrostatic and hydrophobic interactions [182]. The aforementioned inhibition of IAPP conversion into cross- ß fibrils resulted into aggregation due to the presence of strong hydrogen bonds, aromatic stackings as well as salt-bridges between GQDs and the amyloid protein.

As previously mentioned, amyloidosis can also be found in bacterial biofilms. These biofilms consist of structural and functional microbial communities that are also responsible for various severe illnesses [172,173]. Chapman et al. reported the first functional amyloid protein, CsgA, found in the curli fibrils from *E. coli* and other bacteria [183]. Other human microbiomes—such as *Streptococcus*, *Staphylococcus*, and *Salmonella*—have presented the same structural and biophysical properties for extracellular amyloid aggregation found in *E. coli* [171]. Wang et al. studied the antibacterial properties of GQDs with biofilms of *Staphylococcus aureus* [173]. GQDs was determined as a potential therapeutic agent for the disassembly of such biofilms due to their high ROS production, membrane breakage, and their small size [173].

An additional clinical application of GQDs is the photothermal therapy (PTT) for cancer treatment. PTT is a non-invasive substitute for conventional cancer therapy that operates with the use of laser light and electromagnetic radiation distributed into the contrast material applied to the cancer region causing thermal damage [184]. The presence of large sp^2^ carbon agglomeration on the GQDs surface and the carboxyl groups attached around the edges enable the incorporation of highly aromatic drug molecules using the π–π interactions of the GQDs [185]. Li et al. demonstrated that GQDs presenting these above-mentioned characteristics show great water solubility and suitability for conjugation with ligands such as folic acid (FA) and an outstanding load-bearing capability for theranostic agents, namely IR-780 iodide, which renders the GQDs the ideal candidate for PTT and targeted tumor imaging [186].

With the continuous development of nanotechnology for biomedical applications, the incorporation of graphene-based nanomaterials in photodynamic therapy (PDT) has exhibited great therapeutic improvement [187]. PDT can be used as an oncologic intervention that involves oxygen and the exposure of photosensitizers (PSs) to specific wavelengths of light in order to damage cancer tissue [188] as well as an antimicrobial technique to eliminate bacteria by light irradiation [189]. The PS molecules absorb the emitted light—i.e., ultraviolet (UV) or visible (VIS)—which promotes them from the ground state to an excited triplet state passing through an intermediate singlet state, resulting in the generation and release of ROS, such as the singlet oxygen (^1^O_2_), at the targeted cell [190]. However, some PS molecules may undergo a fluorescent process in which they fall from the excited state to a lower energy state by releasing a photon [69]. These fluorescence properties of some PS have been used for real-time medical detection and bio-imaging of various diseases such as cancer, Alzheimer’s, etc. [69,108]. These multi-functional PSs are known as theranostic agents, as they work for both medical diagnostics and therapy. Even though many clinical advantages have been attributed to PDTs such as minimal invasion, non-cumulative toxicity and extraordinary functional and medical results, some limitations on selecting the most adequate photosensitizer and the appropriate light intensity may difficult their effective application for clinical treatments [187]. Currently, PSs do not only attack the targeted malignant cells, but as light irradiation must be implemented for target treatment, this can also generate ROS in both normal and healthy cells, damaging them irreparably [191]. Nevertheless, the use of GQDs may assist on overcoming the previously mentioned challenges of choosing the ideal PS due to their excellent catalytic activity, genuine fluorescence properties, and good photo stability [192].

According to a study by Tabish et al., GQDs can be engineered to be cyto-compatible in PDT applications considering its promising singlet oxygen generation ability while it can be synthesized with high QY that support their PDT application. GQDs are confirmed to have impressive electron conducting network, photo-stability, corrosion resistance, as well as water dispersibility and pH stability that are useful for PDT applications [103]. The simplification of the principle behind killing the tumors by PDT is stemming from the fact that a photo-activated PSs can generate singlet oxygen inside the tumor cell which results in oxidative stress generation and cell death. ROS generation is mainly dependent on the type of the graphene and the route of their administration [108]. For instance, Zhou et al. showed that when GQDs lack different groups—such as carboxyl, ketonic carbonyl, or hydroxyl—their ROS generation ability varies as well and GQDs generate ROS in correlation with the oxygen content of the GQDs structure. As with their photostability, ROS generatability was affected from this situation [193]. Ahirwar et al. showed that the breast cancer cells were killed by 90% only in 5 min of exposure due to singlet oxygen generation when they employed GQDs as PSs [68]. Li et al. demonstrated that fluorine containing GQDs can generate more singlet oxygen QY compared to pristine GQDs indicating better PDT agent characteristics. Zhang et al. produced a PDT agent with GQD-decorated up-conversion nanoparticles (UCNP-GQD) using tetramethylrhodamine-5-isothiocyanate (TRITC) as mitochondria-targeting ligand for therapeutic cancer treatment [194].

GQDs show excellent properties as electron donors as well as electron acceptors and at the same time functioning as a theranostic agent and an outstanding source for ROS [160]. Furthermore, GQDs are able to generate cytotoxic ROSs by converting biomolecules from normal species such as ^3^O_2_ to ^1^O_2_ via electron transfers and light exposure makes them an exceptional PDT agent with the highest QY reported so far (QY > 1.3) [159,195,196]. In addition, the free radicals present at the GQDs surface and the capacity to transfer energy directly into oxygen molecules are considered the main causes in the generation of high amounts of ^1^O_2_ [196]. Kuo et al. reported the use of N-GQDs as a PS to eradicate microbes, in this case the bacterium *E. coli*, and to replace antibiotic treatments using PDT [188]. This work exhibited the promising electrocatalytic, photochemical, and electrochemical properties of N-GQDs as well as their capacity of producing more ROS, which are responsible for enhancing the PDT effect.

## 4. GQDs as Potent Electrode Material for the Development of Micro-Supercapacitors (MSCs)

Considering the increased demand for wearable and portable electronics such as flexible displays and electronic textiles, the advancement in flexible energy-storage devices has now become highly crucial and interesting [197,198,199]. In the last few years, owing to a high demand for lightweight and flexible portable energy storage devices, electrochemical capacitors or supercapacitors have garnered enormous attention and have exposed tremendous potential for portable electronic devices [200,201,202,203,204]. They are utterly distinct from the conventional capacitors and can fill the void between lithium-ion batteries and traditional capacitors. In fact, supercapacitor materials serve the merits of both rechargeable batteries as well as dielectric capacitors, which make them ideal candidates to acquire operational safety, fast charge–discharge ability, long shelf-life, low maintenance cost, and high power density [205,206,207,208].

To attain wearability and portability, the architecture of flexible power sources is quite significant for designing wearable micro-supercapacitors (MSCs) [209]. Flexible fiber supercapacitors (FFSCs) are being studied as one of the most trustworthy flexible power sources due to their extended cycling time, easy operation, quick charge–discharge rate, and enhanced power delivery. In addition, FFSCs are versatile because of their distinct wire-shaped assembly and can be readily altered in accordance with the requirement to develop MSCs [209,210].

MSCs can be classified on the basis of how the active components reserve the energy. They can be either electric double layer capacitance (EDLC) supercapacitors or pseudo-capacitors. The former works on storage of charge via physical ionic deposition, whereas the latter via redox reactions [211]. In recent years, various materials such as composite materials [212], carbon-materials [213], metal oxides [214], and conducting polymers [215,216] have been established as electrode materials for the fabrication of micro-supercapacitors. Among these, carbon-based micro-supercapacitors have been extensively studied because of their wide pore size distribution, excellent conductivity, large surface area, as well as high cycle stability of carbon electrode materials [45,217,218,219,220,221,222,223,224]. The majority of carbon electrode materials include carbon fibers (CFs), graphene, CNTs, carbon dots, and GQDs [225,226,227,228].

GQDs serve as an electrode material for MSCs, particularly due to their excellent quantum confinement and edge effects. Highly doped GQDs (e.g., C-, N-, and O-doped GQDs) could provide a high concentration of active sites due to the co-doping as well as most marked edge effect, which can result in higher specific capacitance [229,230]. In addition, graphene sheets (GS) are also considered as a good electrode material for high-performance supercapacitors [231,232]. When compared to GS, GQDs possess nanometer-size, better solubility in wide range of solvents, and abundant edge defects, making them more appropriate for developing micro-supercapacitors [233,234].

For flexible all-solid-state supercapacitors, N-GQDs can act as remarkable pseudo-capacitive materials when assembled on carbonized metal–organic framework (MOF) materials integrated with CNTs to synthesize hierarchical 3D all-carbon electrode materials [235]. In such a system, CNTs and carbonized MOF materials provide electrical conductivity and higher surface area, respectively. It was reported that, owing to the combined additive effect of the aforementioned components, the resulting micro-supercapacitor could offer energy density of 18.75 Wh kg^−1^ with a power density of 108.7 W kg^−1^ and high specific capacitance of 540 F g^−1^ at a current density of 0.5 A g^−1^ [235]. The greater edge-to-core atomic ratios exhibited by N-GQDs could enhance electrolyte wettability. This design could be able to operate economic light emitting diodes for about 20 min, which directs a new path for high-performance energy storage fields [235].

**Figure 11:**
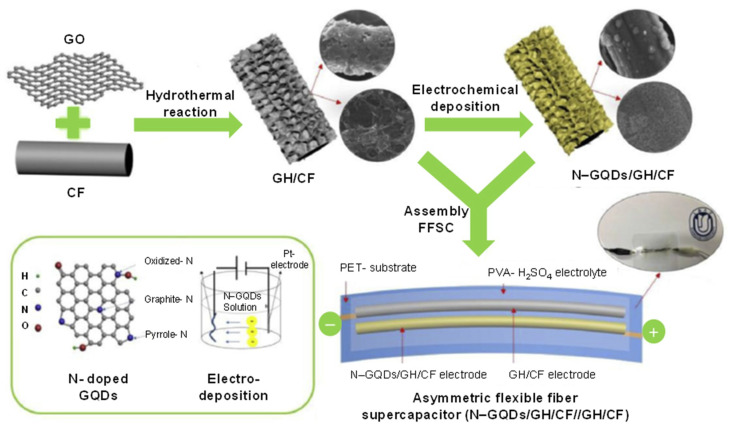
Schematic representation of the fabrication process of the N-GQD/GH/CF positive electrode and GH/CF negative electrode and their assembly into an asymmetric FFSC [209].

Carbon-based FFSCs are favorable power sources for wearable and portable electronic devices. A study reported the synthesis of N-GQDs/CF hybrids using graphene hydrogel (GH) [209]. This GH could serve as a 3D porous buffer layer, which was implanted on CF to obtain ternary carbon hybrid of N-GQD/GH/CF (Figure 11). Herein, N-GQDs with high pseudo-capacitive performance were electrochemically deposited on the GH/CF layer. Such a micro-supercapacitor could provide a volumetric capacitance of 93.7 F cm^3^ at 20 mA cm^3^, which was almost 7-fold more than that of the GH/CF, whereas the capacitance retention reached 88% after 5000 cycles. The improved performance and excellent flexibility of FFSCs can be attributed to the presence of N-GQDs in such a system [209].

Currently, mechanically flexible as well as optically transparent energy storage appliances are being studied and developed owing to their high ability to act as integrated power sources [210]. Lee et al. designed such a flexible and transparent micro-supercapacitor based on GQDs and chelated graphene using the electrophoretic deposition (EPD) technique. In this work, GQDs were robustly incorporated on an interdigitated framework of monolayer graphene due to the formation of chelate between GQDs and graphene with metal ions. The subsequent resultant pattern was employed as the potent electrode material for micro-supercapacitor. This device could exhibit a high energy storage and excellent transparency of 9.09 μF cm^−2^ and almost 93%, respectively [210].

N-doped porous carbon synthesized by implanting N-GQDs onto a carbonized metal–organic framework (cMOF-5) can serve as an electrode material for micro-supercapacitors, where N-GQDs offers enhanced pseudo-capacitance as well as increased surface wetting ability and cMOF-5 offers a cubic porous pattern with a higher electro-conductivity as well as greater specific surface area. Such an N-GQD/cMOF-5 electrode material could generate a specific capacitance of 294.1 F g^−1^ at 0.5 A g^−1^ and 780 F g^−1^ at 10 mV s^−1^ in a three-electrode system [39].

**Figure 12:**
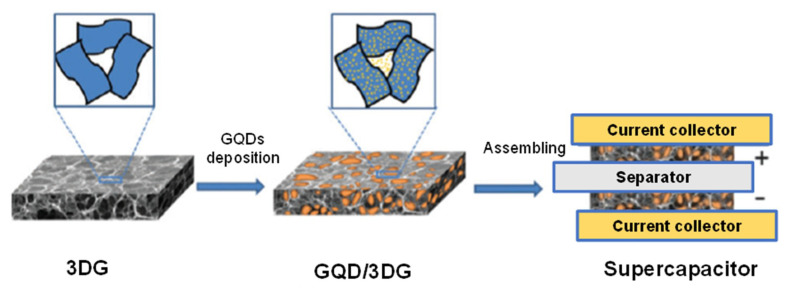
Schematic illustration of a GQD/3DG based symmetrical micro-supercapacitor [234]. Reproduced by permission of The Royal Society of Chemistry.

Combining 3D graphene (3DG) framework with other functionally active components increases the overall ability of micro-supercapacitors [236]. As presented in Figure 12, GQDs were electrochemically incorporated onto the 3DG framework. The researchers reported that such a highly stable MSCs system engineered from the GQD/3DG could exhibit high specific capacitance of 268 F g^−1^, which was 90% improved than those with only 3DG electrodes [234].

Ouyang et al. reported a hydrothermal top-down synthetic approach for the production of S, N-GQDs, which could exhibit a high specific capacitance of 362.60 F g^−1^ at a fixed scan rate of 5 mV s^−1^. This high performance was resulted from the pseudocapacitance offerred by S and N. This doping could improve the charge storage capacity [237]. Mondal et al. synthesized GQD-doped polyaniline fibrous composites, which could show good cyclic stability and a retention life time of 80.1% after 3000 cycles. It was reported that these as-prepared GQDs exhibited specific capacitance value of about 1044 F g^−1^ at a current density of 1 A g^−1^ [238]. GQDs with size < 5 nm when prepared by top-down approach using nitric acid (HNO3) as a reaction precursor could show high electrochemical performance for the development of MSCs [239]. Such a GQDs-based system could provide an ideal EDLC features including high cycling stability, negligible relaxation duration, small internal resistance, good energy density of 41.2 W h kg^−1^ at 1 A g^−1^ and an excellent specific capacitance of 296.7 F g^−1^. These characteristics demonstrated the enhanced electro-conductivity and improved ion transferability [239].

Generally, carbon-based micro-supercapacitors working on the principle of EDLC are limited by the energy loading densities and quite low specific capacitances and energy, since the corresponding electrode materials are derived from weakly active and huge carbon components [240]. To overcome this drawback, strongly active N and O co-doped GQDs can be used to obtain abundant active sites, increased hydrophilicity and extremely smaller sizes [240]. In a study reported in 2018, N-O-GQDs MSCs could offer higher volumetric capacitance of 325 F cm^3^ in sulfuric acid owing to their elevated electrolyte wettability, high storage density, and excellent pseudo-capacitance property [240].

Tjandra and colleagues reported that GQDs can also be combined with materials to fabricate freely placed and flexible EDLC supercapacitor electrode [211]. Herein, hydrothermally synthesized GQDs were electrodeposited on carbon cloth to improvise its capacitance up to 70 mF cm^−2^ without any significant mass charging. Another EDLC supercapacitor electrode by electrodepositing GQDs, but on CNTs provided an enhanced capacitance of 44 mF cm^−2^, which was around twice as high as just using CNTs [233].

**Figure 13:**
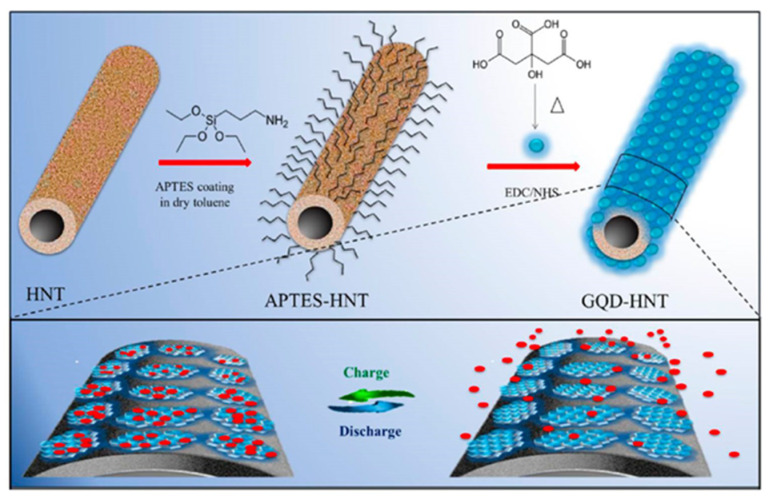
Synthesis of GQDs/HNTs hybrid for the fabrication of micro-supercapacitor electrode [241].

Halloysite nanotubes (HNTs) that are rich in active sites for storing energy, when combined with GQDs can be employed for the development of eco-friendly supercapacitors. Ganganboina et al. reported such a device using GQDs/HNTs nanomaterials (Figure 13) in the co-existence of APTES (3-aminopropyl-triethoxysilane) to offer rapid charge transfer and accelerated charge storage sites [241]. Moreover, it could provide specific capacitance of 363 and 216 F g^−1^ at current densities of 0.5 and 20 A g^−1^, respectively. In other studies, the same group has developed similar MSCs, but by assembling Fe_3_O_4_ along with GQDs/HNTs to generate even higher specific capacitance (i.e., 418 F g^−1^) [242]. The Fe_3_O_4_/HNTs could enhance the active sites for charge storage by reducing the diffusion path of electrons. Table 3 highlights the specific capacitance values corresponding to some more GQD-based supercapacitors.

## 5. Conclusions

GQDs are confirmed to be excellent imaging probes by numerous applications with their small sizes. After the first realization of their photoluminescence feature, studies were shaped towards the highly qualified, non-toxic, non-invasive, and target-oriented bio-imaging and therapy applications. It is possible to manufacture GQDs in greater amounts than the laboratory conditions by preparing industrial-scale productions, without dissipating any of their distinctive features that enable the bio-imaging. Moreover, the abundant carboxylic groups present in GQDs allows their functionalization with active biomolecules and are widely used for the therapy development and drug delivery. Various methods for the synthesis of GQDs have been updated day by day with increasing quality and efficiency. On the other hand, doping GQDs with diverse atoms endow unique attributes to these tiny particles. Hence, it is very likely and preferred that GQDs are holding an immense promise for a wide range of bio-imaging studies as well as commercialized applications. Moreover, as a potent electrode material for the fabrication of micro-supercapacitors, GQDs offer benefits for developing portable and highly efficient electronic tools owing to their quantum confinement and edge effects.

## Figures and Tables

**Table 1:** Various approaches for synthesizing GQDs with their respective size ranges and QY.

Modification	Source/Precursor	Synthesis Method	Size	QY	Reference
A-GQDs	Graphite and adenine precursors	Two-step microwave-assisted method	3–5 nm	21.63%	[61]
Hg-PEHA-GQD-DPA	Citric acid	Two-step continuous thermal pyrolysis	3.16 nm	90.91%	[62]
RF-GQDs	Graphite in K_2_S_2_O_8_ solution	Electrochemical exfoliation	3 nm	1.8%	[63]
B-GQDs	4-vinylphenylboronic acid and boric acid	-	5.8 nm	11.2%	[23]
(GQD/DBM)_3_EuPhen/GQD	Graphite rod	Electrochemical exfoliation	5.5 ± 0.4 nm	15.5%	[64]
sGQDs	Ethanolic extract of grape seed extract powder	One-pot microwave-assisted synthesis	~50–60 nm	31.79%	[30]
DOX-GQD-RGD	Thermally exfoliated graphite oxide	Refluxing with concentrated nitric acid	3.7 nm	-	[65]
GQDs	Trisodium citrate	Pyrolytic carbonization route	1.3 ± 0.5 nm	3.6%	[9]
mGQDs	Mango leaves	One-pot microwave-assisted green-synthesis route	2–8 nm	-	[66]
HD-GQDs, LD-GQDs	Acetylene black, nano-graphite	Ultrasonic-assisted liquid-phase exfoliation technique	2–6 nm, 2–9 nm	1.8%, 2.4%	[67]
GQDs	Graphite rod	Electrochemical exfoliation method	1.5–5.5 nm	-	[68]
F-GQDs	Fluorinated graphite	Oxidative cutting method	2.1 nm	13.72%	[69]
GQDs	Pyrene precursor	Wet chemistry treatment of commercially available polycyclic aromatic hydrocarbon	5–10 nm	11.7%	[70]

Abbreviations: A-GQDs: Adenine-modified GQDs; B-GQDs: Boron-modified GQDs; DBM: Dibenzoylmethane; DOX: Doxorubicin; DPA: D-penicillamine; Eu: Europium; F-GQDs: Fluorinated GQDs; HD-GQDs: High defect GQDs; Hg: Mercury; K_2_S_2_O_8_: Potassium persulfate; LD-GQDs: Low defect GQDs; m-GQDs: *Magnifera indica* derived GQDs; PEHA: Pentaethylenehexamine; Phen: Phenanthroline; RF-GQDs: Red fluorescent GQDs; RGD: Arginine-glycine-aspartate; s-GQDs: Self-assembled GQDs; QY: Quantum yield.

**Table 2:** GQDs and their cytotoxic effects.

GQD-Type	Cytotoxicity	Media	Notes	References
GQDs	GQDs have minimal dark toxicity	In vivo toxicity in rats	Some minor changes were particularly noted in the liver and lungs at the 10 and 15 mg/kg doses of GQDs	[103]
Carboxylated GQD	No acute cytotoxicity between the range of 5 and 10 mg/kg	Liver, spleen, kidney, and tumor/In vivo and in vitro	Accumulation in mice liver, spleen, kidney, and tumor at 24 h after intravenous injection of GQDs	[111]
GQDs	No severe toxicity in mice with 300 micrograms of GQDs (per head, 15 mg/kg)	Intraperitoneal infusion to mice	GQDs could be excreted out of the body	[104]
GQDs	Low toxicity	Blood derived CD^34+^ cells from leukapheresis	SEPW1 is downregulated with a fold change of −5	[107]
GQDs	Very low cytotoxicity	B16F10 cells and MCF-7 cells		[68]
GQDs	Low cytotoxicity to 0–400 µg mL^−1^ GQDs for 24 h	HeLa	More than 80% cell survival rate	[9]
GQDs	No overt acute toxicity	Lung tissues of rats	In high dose group alveolar septa thickening during inflammation	[100]

**Table 3:** Examples of MSCs involving the use of GQDs with their respective capacitive performance.

Electrode Material	Specific Capacitance	References
Amine-functionalized single-crystalline GQDs	400–595 F g^−1^	[243]
Nitrogen and oxygen co-doped GQDs/carbon nanotubes/carbon cloth	212 F g^−1^	[244]
GQDs/NiCo_2_S_4_ with hierarchical-like hollow nanostructure	678.22 F g^−1^	[245]
GQDs/MnO_2_	1170 F g^−1^	[246]
Ultra-microporous carbons integrated GQDs	270 F g^−1^	[247]
GQDs/polypyrrole	485 F g^−1^	[248]
Cobalt (II) chloride–GQDs	~300 F g^−1^	[249]
Fe (II)S–GQDs	476.2 F g^−1^	[250]
Porous carbon nanosheets/GQDs	230 F g^−1^	[251]
